# Global trends in *BRCA*-related breast cancer research from 2013 to 2022: A scientometric analysis

**DOI:** 10.3389/fonc.2023.1197168

**Published:** 2023-07-05

**Authors:** Yujie Huang, Daitian Zheng, Qiuping Yang, Jinyao Wu, Huiting Tian, Zeqi Ji, Lingzhi Chen, Jiehui Cai, Zhiyang Li, Yexi Chen

**Affiliations:** Department of Thyroid, Breast and Hernia Surgery, General Surgery, The Second Affiliated Hospital of Shantou University Medical College, Shantou, Guangdong, China

**Keywords:** *BRCA*, breast cancer, scientometric analysis, CiteSpace, VOSviewer

## Abstract

**Introduction:**

Since the mid-2000s, breast cancer incidence among women has slowly increased at about 0.5% per year. In the last three decades, Breast Cancer Susceptibility Gene (*BRCA*) has been proven to be the crucial gene in encouraging the incidence and development of breast cancer. However, scientometric analysis on *BRCA*-related breast cancer is in shortage. Thus, to have a clear understanding of the current status and catch up with the hotspots, a scientometric analysis was conducted on specific academic publications collected from the Web of Science (WoS).

**Methods:**

We searched the Web of Science Core Collection (WoSCC) to procure associated articles as our dataset. Bibliometric, CiteSpace, VOSviewer, and HistCite software were then applied to conduct visual analyses of countries, institutions, journals, authors, landmark articles, and keywords in this research field.

**Results:**

A total of 7,266 articles and 1,310 review articles published between 2013 to 2022 were retrieved eventually. The annual output steadily rose year by year and peaked in 2021. The USA led the way in the number of published works, total citations, and collaboration. *Breast Cancer Research and Treatment* was the most favoured journal in this research field. Narod SA from the University of Toronto produced the most publications. At last, the most prominent keywords were “breast cancer” (n=1,778), “women” (n=1,369), “*brca1*” (n=1,276), “ovarian cancer” (n=1,259), “risk” (n=1,181), and “mutations” (n=929), which exposed the hotspots within the *BRCA* domain of breast cancer study.

**Conclusion:**

The tendency in the *BRCA* research field over the past decade was presented by the scientometric analysis. The current research focus is the clinical trials of poly-adenosine diphosphate ribose polymerase inhibitors (PARPi) drugs and their resistance mechanisms.

## 1 Introduction

The World Health Organization (WHO) estimated that by 2021, breast cancer would be the most prevalent worldwide, accounting for 12% of all yearly new cancer cases. In addition, women with breast cancer globally lose more disability-adjusted life years (DALYs) than any other kind of cancer. With a population of 0.42 million, breast cancer was the most common type of cancer that killed women in China (1). The risk factors that weigh heavily in breast cancer can be divided into two aspects: I. Genetic factors: family history of breast cancer, inheritance susceptibility, et al. II. Non-genetic factors: sex, age, diet, exercise, weight, alcohol assumption, benign breast cancer, early pregnancy, breastfeeding, menopause, hormone replacement therapy, et al. (2–5). For the general population, sticking to a healthy lifestyle would be a good approach to preventing breast cancer incidence. However, for those high-risk individuals significantly driven by genetic factors, the options for prevention and treatment are still in limitation (3, 5). Ultimately, breast cancer commonly metastasizes to other organs in the terminal stage, resulting in a bleak prognosis (6). In the face of the tricky situation of breast cancer, it is pressed for further studies and effective treatment.

Among breast cancer-mutant genes, the most common genes are Breast Cancer Susceptibility Gene 1 (*BRCA1*) and Breast Cancer Susceptibility Gene 2 (*BRCA2*) involved in DNA repair (3, 7). *BRCA* is a cancer suppressor gene, including *BRCA1* and *BRCA2*, identified in 1990 and 1994, respectively (5). Both of them encode large proteins involved in DNA repair and recombination, cell cycle control, chromatin modulation, checkpoint enforcement, and transcription. In DNA repair, *BRCA1* and *BRCA2* particularly serve as components of the DNA damage response pathway. When DNA single-strand break (SSB) occurs, the base-excision repair (BER) is activated. Poly-adenosine diphosphate ribose polymerase (PARP) is the key enzyme involved in BER. It could bind to damaged DNA at sites of SSB, thereby leading to the recruitment of DNA repair effectors to sites of DNA breaks (6, 8, 9). Mutations in *BRCA* will inhibit wrong DNA from being repaired correctly and finally contribute to cancer. In *BRCA1* carriers, breast cancer (72.5%) ranked first by the cumulative risk, followed by ovarian cancer at 65.6% and gastric cancer at 21.3%. Similarly, for *BRCA2* carriers, the highest cumulative risk was also breast cancer (58.3%), and the next two were prostate cancer (24.5%) and gastric cancer (19.3%) (10). The female population with *BRCA1/2* mutations has several options to prevent developing breast cancer, including surgery, medication, and lifestyle (5). According to research, high-risk women with a bilateral risk-reducing mastectomy (BRRM) had a 90% lower risk of developing breast cancer. BRRM usually served as an intervention choice among *BRCA* carriers, especially for *BRCA1* carriers (3). While in the treatment of *BRCA1/2*-mutant breast cancer, it continues to involve highly invasive surgical procedures like most cancers (11). For prevention or treatment purposes, the impact on psychology and life induced by mastectomy can not be ignored. Since the approval of poly-adenosine diphosphate ribose polymerase inhibitors (PARPi) in *BRCA1/2* mutant breast cancer in 2018, it has served as a risk-reducing medication and prolonged the prognosis-free survival distinctly. PARPi drugs exert their therapeutic effects by binding with the PARP on DNA and preventing it from being released from DNA break sites, thus, blocking the wrong DNA damage repair. However, resistance to PARPi is inevitable in therapy, and it is urgently needed to overcome it (9). On the other hand, expanding the profiting population of PARPi and reducing adverse effects are also imperative nowadays (12, 13). Overall, research on *BRCA*, especially on targeted treatment, is far from enough compared with its encouraging prospects. Considering the current situation, we carried out this scientometric analysis to summarize previous publications and suggest further research directions.

This paper is structured as follows: Section one is the Introduction and presents the current situation of *BRCA* and breast cancer together with the objectives of this paper. Section two Methodology: searching and screening strategies, as well as visualization of countries, institutions, authors, references, and keywords using specific software. Section three Results: this section shows the results of the following analyses, they are annual growth trend of publications analysis, countries/regions and institutions analysis, journals, publishers and research areas analysis, authors analysis, articles and references analysis, and keywords analysis. Section four are Discussion and Conclusion.

## 2 Methods

### 2.1 Data collection

The Web of Science (WoS) was chosen as the data source for this investigation. Many scholars recognize WoS as a top-notch database of digital literary resources, and it is often utilized in bibliometric analysis (14, 15). On January 16, 2023, we searched for related publications in the field of *BRCA* in breast cancer through the Web of Science Core Collection (WoSCC) in the Science Citation Index Expanded (SCI-EXPANDED)—2003-present Edition. The medical entry terms “*BRCA*” and “breast cancer” served as central words, and detailed searching strategies include the following: “breast cancer*” (Topic) or “breast neoplasm*” (Topic) or “breast tumor*” (Topic) or “breast carcinoma*” (Topic) or “mammary cancer*” (Topic) or “mammary carcinoma*” (Topic) or “mammary neoplasm*” (Topic) AND “BRCA*” (Topic) or “brochocin-C*” (Topic) or “*brcB protein*, Brochothrix campestris*” (Topic) or “*brcA protein*, Brochothrix campestris*” (Topic). Moreover, these publications were then limited to being published between 2013 and 2022. We also set restrictions on the document type and language: only articles and review articles in English were extracted. After that, five articles were published in 2023, and two retracted articles were excluded. In sum, 8,576 publications were filtered out from a large number of search results: 7,266 articles and 1,310 review articles, respectively. Finally, full records and cited references of the 8,576 publications were exported in the plain text file for future analysis. Besides, to remove bias, the work of searching and downloading was completed in one day.

### 2.2 Statistical analysis

Bibliometric analysis is usually applied to analyze research results with mathematical and statistical methods, to obtain adequate information and reveal the macroscopic development law of a great variety of publications. Through bibliometric analysis, detailed information about countries, institutions, journals, authors, keywords, and references can be easily obtained (16). Furthermore, through visualization, we can thoroughly assess the state of the research progress in a specific scientific field and predict the trends and hotspots of a certain field (17–20). CiteSpace, VOSviewer, and HistCite are the three most often used bibliometric analysis tools, according to research by Xuelian Pan et al. into the usage and dissemination of bibliometric mapping software in 2018 (21). The detailed statistical analysis process is shown as follows.

The build-in function from WoS called “analyze results” and “citation reports” were preliminarily employed to obtain information on the year of publication, literature type, research area, author, affiliated institution, journal, publisher, country, language, funding agency, and open access of these publications. Through the “Citation Report” function of WoS, we obtained the total number of cited articles and the number of non-self-cited articles, the sum of cited times and non-self-cited citations, h-index, et al. The data collected from WoS contains titles, authors, publishers, languages, document types, keywords, abstracts, affiliations and cited references, which were then imported into Biblioshiny (a web interface for bibliometric), VOSviewer (1.6.18), CiteSpace (6.1.R2) and HistCite Pro (2.1) for scientometric analysis.

Biblioshiny is a tool designed in R to be versatile and to have easier integration with other graphical and statistical tools (22). The collected data ranging from authors to cited references, was imported into the Biblioshiny as raw files. The main information on the publications was acquired through Biblioshiny, including an overview of all publications, annual scientific production, countries/regions, institutions, local source impact, authors, word cloud, et al.

VOSviewer can visualize the research profile of authors, references, countries, et al. by showing how their output and impact are distributed over scientific fields (23). Co-occurrence analysis could be performed on countries/regions, organizations, authors, and keywords by different methods such as network visualization, coverage visualization, and density visualization. VOSviewer was used to explore collaboration networks of countries and authors, co-citation authors, and references, thus, bringing a clear relational graph for us.

**Figure 1:**
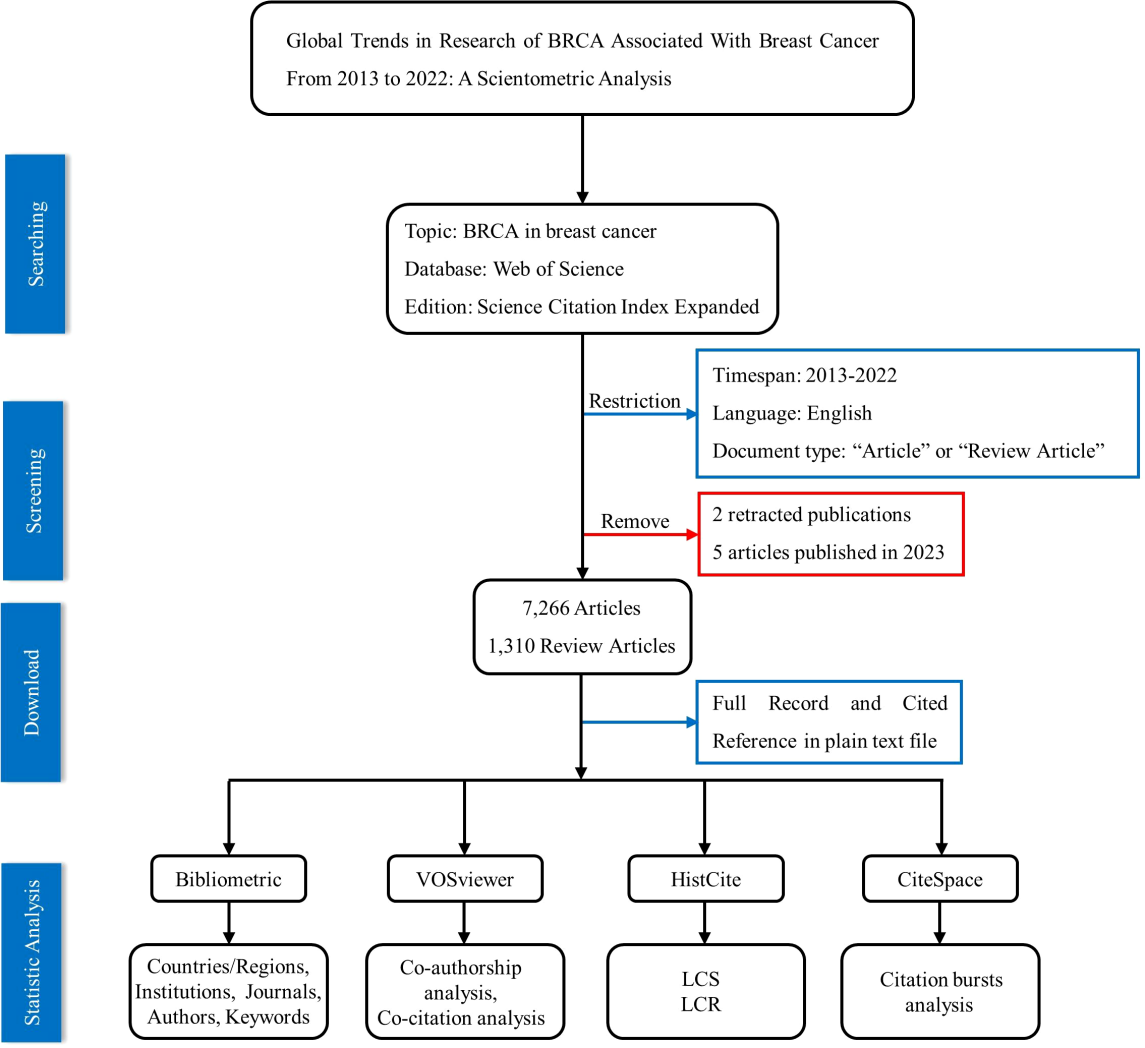
Flow chart of the data collection and screening process for the statistic analysis.

The third software is CiteSpace; it is a self-signed Java application (24) which was applied to have a list of the top references and keywords with the strongest citation bursts. Finally, to figure out the important research results in this field, we employed HistCite Pro to seek out the most locally cited articles and the most cited references for retrieved articles analysis. The data collection and statistical analysis process are concisely shown in **Figure 1**.

## 3 Results

### 3.1 Overview of the main information

Through the information offered by Biblioshiny and combined with the “analyze results” (WoS built-in function), we made a preliminary determination as to whether the outcomes satisfied the all-inclusive standards. We sorted out the data source and some descriptive statistics in a form as shown in **Supplementary Table 1**. The average age of the articles was 4.98 years, and the average number of citations per article was 22.89. Of the 8,576 publications, the overall cited times were 196,280 (42,207 self-citations). It was noteworthy that the international co-authorship (%) reached 28.42%, indicating that the research field has formed a good collaboration network between countries/regions.

### 3.2 Annual growth trend of publications

**Figure 2:**
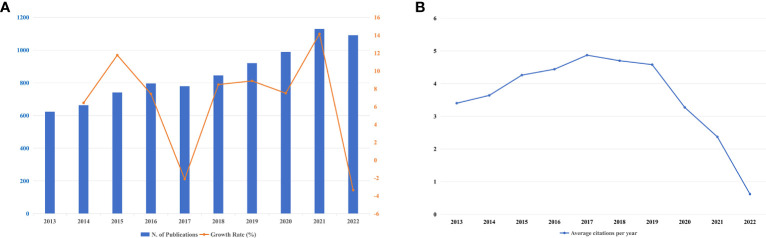
**(A)** Annual publications output and corresponding growth rate between 2013-2022. **(B)** The number of average citations per year in *BRCA* associated with breast cancer.

Throughout the past ten years, 8,576 publications have been published on this subject altogether. **Figure 2A** depicts the annual output (r=0.977; *p*<0.001) and corresponding growth rate between 2013-2022. In **Figure 2A**, the blue bar refers to the annual scientific output, and the orange broken line portrays the growth rate. Besides, the X-axis of **Figure 2A** shows the years from 2013 to 2022, whereas the number of publications is indicated on the left Y-axis. The general pattern of the number of publications every year was an upward trend but except for a little decline in 2017 and 2022, and it rose from 623 in 2013 (accounting for 7.26%) to a peak of 1,129 in 2021 (accounting for 13.16%). The average annual growth rate of scientific output was 6.57%, peaking in 2021 at 14.16% and falling to -3.3% in 2022. Citation analysis helps to judge the trustworthiness of a long list of publications, and the number of cited times reflects its scientific impact. **Figure 2B** states the average citations per year, it is easy to tell that the average citations had a steady rise from 2013 (citations=3.4) to 2017 (citations=4.87), but from 2018 (citations=4.7) to 2022 (citations=0.62), it was an irresistible downtrend. **Supplementary Table 2** itemizes annual citations covering a period from 2013 to 2022. The highest mean total citations per article fell in 2013 (citations=37.35), and the lowest was in 2022 (citations=1.25). The year 2017 had the most citations on average (citations=4.87), while 2022 had the fewest (citations=0.62).

### 3.3 Countries/regions and institutions analysis

**Figure 3:**
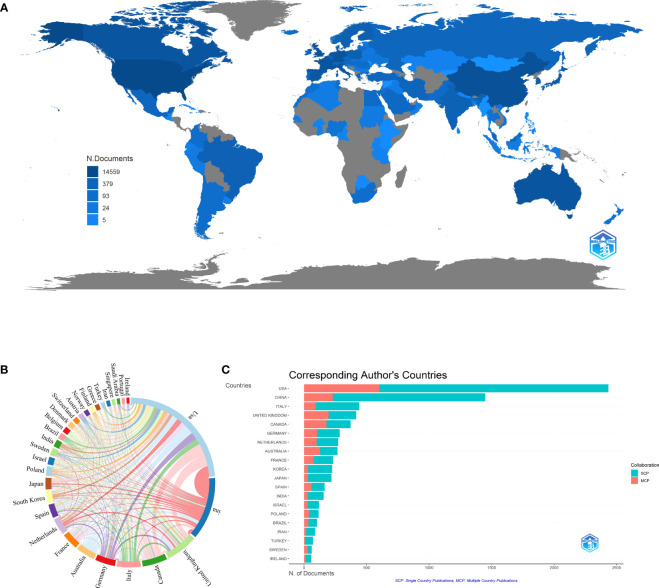
**(A)** Distribution of publications from different countries/regions in *BRCA* associated with breast cancer. **(B)** The collaboration network between countries in *BRCA* associated with breast cancer. **(C)** Top 10 countries’ publications partnerships in *BRCA* associated with breast cancer.

There were 105 countries engaged in the *BRCA* research field, as shown in **Figure 3A**. The countries/regions refer to the corresponding authors’ location, while the depth of the blue color is related to its production: the higher production, the bluer color. It was easy to find that the USA, China, Canada, and some European countries possess a deeper color. Meanwhile, we listed out the top ten countries with the most scientific production in **Table 1**. The leading country was the USA (n=2,434, 28.38% of the publications) with the maximum number of publications, followed by China (n=1,449, accounting for 16.90% of the publications), and Italy (n=440, accounting for 5.13% of the publications). It was worth noting that China was the only developing country on the list. The major participants included five European countries, two North American countries, two Asian countries, and one Australian country. However, the United Kingdom had the greatest average article citations (citations=55.82), far higher than the USA (citations=32.19). By contrast, China had the second most publications but a relatively low average of article citations (citations=11.85). Co-authorship analysis of countries measures cooperative links, which are defined by the number of documents that are co-authored. To visualize the collaboration network of the top 30 most productive countries, we applied VOSviewer and Microsoft Charticulator to form a chord diagram, as shown in **Figure 3B**. Each country is represented by a certain type of color block, and the size corresponds to the number of publications. As for the line between countries, its thickness mirrors the degree of cooperation closeness. The collaboration line of the USA (total link strength=3,047) accounted for nearly one-quarter of the diagram, indicating that it was the country with the highest level of cooperation. The United Kingdom ranked second (total link strength=2,034) and was followed by Canada (total link strength=1,549), Australia (total link strength=1,430), and Germany (total link strength=1,389). **Figure 3C** illustrates the publications partnerships between the top ten countries in the research field of *BRCA* associated with breast cancer. SCP refers to Single Country Publications, while MCP is Multiple Country Publications, and they denote the number of papers co-authored by authors of the same nationality and different nationalities. We can judge from the figure that international cooperation was quite high, which was consistent with **Figure 3C**. However, the number of publications would affect the quantity of MCP, so the MCP ratio (MCP/SCP) was induced to evaluate the collaboration level, as shown in the last row of **Table 1**. Canada owned the highest MCP ratio (0.481), followed by Australia (0.472) and the United Kingdom (0.47).

**Table 1:** Top 10 most productive countries/regions in the research filed of *BRCA* associated with breast cancer.

Rank	Countries/Regions	Publications	% of 8,576 publications	Total Citations	Average Article Citations	MCP/SCP
1	USA	2,434	28.38%	78,352	32.19	0.247
2	China	1,449	16.90%	17,168	11.85	0.159
3	Italy	440	5.13%	7,584	17.24	0.211
4	United Kingdom	417	4.86%	23,278	55.82	0.470
5	Canada	372	4.34%	9,257	24.88	0.481
6	Germany	285	3.32%	7,433	26.08	0.361
7	Netherlands	272	3.17%	6,669	24.52	0.368
8	Australia	267	3.11%	6,016	22.53	0.472
9	France	232	2.71%	4,788	20.64	0.332
10	Korea	224	2.61%	2,609	11.65	0.143

SCP, Single Country Publications; MCP, Mutiple Country Publications.

In terms of institutions, 8,555 institutions contributed to the retrieved articles. The top 10 institutions with the most publications are mentioned in **Table 2**. Topping the list was the University of Toronto, with 611 publications, accounting for 7.12% of 8,576 publications. The University of Melbourne (n=610), the University of Texas MD Anderson Cancer Center (n=547), and the Netherlands Cancer Institute (n=526) followed closely. There were four institutions located in the USA and two located in Canada. Likewise, VOSviewer and Microsoft Charticulator were used to form a collaboration network between institutions, as shown in **Supplementary Figure 1**. Each color block represents a country; the size is directly proportional to the number of publications. Inter-institution cooperation is represented in the lines connecting the different color blocks. Among the top 30 most productive authors, the University of Toronto (total link strength=367) had the most connections, followed by the University of Melbourne (total link strength=334), Dana-Farber Cancer Institute (total link strength=299), and Stanford University (total link strength=293).

**Table 2:** Top 10 most productive institutions in the research field of *BRCA* associated with breast cancer.

Rank	Institution	Country	Publications	% of 8,576 publications
1	University of Toronto	Canada	611	7.12%
2	University of Melbourne	Australia	610	7.11%
3	University of Texas MD Anderson Cancer left	USA	547	6.38%
4	Netherlands Cancer Institute	Netherlands	526	6.13%
5	Memorial Sloan Kettering Cancer left	USA	480	5.60%
6	University of Cambridge	United Kingdom	450	5.25%
7	McGill University	Canada	363	4.23%
8	Seoul National University	Korea	345	4.02%
9	Dana-Farber Cancer Institute	USA	333	3.88%
10	University of Pennsylvania	USA	327	3.81%

### 3.4 Journals, publishers and research areas analysis

Out of 1,244 kinds of journals, the top ten most productive journals are listed in **Table 3**. Publication number, proportion, H-index, total citations, and IF/JIF Quartile (2022) of the top journals are also illustrated in the table. The journal *Breast Cancer Research and Treatment* (n=350) received a 4.08% share of total publications, followed by *Cancers* (n=254) with 2.96%, and *PLoS One* (n=177) with 2.06%. The most popular journal, *Breast Cancer Research and Treatment*, had 6,153 total citations with an H-index of 39. The most influential periodical was *Breast Cancer Research* (IF=8.408), followed by *Cancers* (IF=6.575) and *International Journal of Molecular Sciences* (IF=6.208). According to the Law of Bradford, journals can be divided into core, relevant, and non-relevant areas that specialize in this subject. The number of publications in each area is the same, and the ratio of the core area: relevant area: the non-relevant area is 1:n:n^2^ (25). For the purpose of identifying the core journals, we employed the Law of Bradford to generate **Supplementary Table 3**. The list of 25 core journals contributed 2,868 publications and accounted for 33.44%; most of the production started in the year 2013. Of the 25 core journals, *Annals of Oncology* had the highest IF of 51.769.

**Table 3:** Top 10 most productive journals in the research field of *BRCA* associated with breast cancer.

Rank	Journal	Publications	% of 8,576 publications	H-index	Total Citations	IF/JIF Quartile (2022)
1	Breast Cancer Research and Treatment	350	4.08%	39	6,153	4.624/Q2
2	Cancers	254	2.96%	19	2,148	6.575/Q1
3	PLoS One	177	2.06%	33	4,048	3.752/Q2
4	BMC Cancer	160	1.87%	27	2,597	4.638/Q2
5	Scientific Reports	154	1.80%	24	2,002	4.996/Q2
6	Oncotarget	143	1.67%	33	3,537	4.345/Q2
7	Frontiers in Oncology	140	1.63%	17	1,031	5.738/Q2
8	Familial Cancer	124	1.45%	17	1,198	2.446/Q4
9	Journal of Genetic Counseling	104	1.21%	21	1,314	2.717/Q3
10	Breast Cancer Research	99	1.15%	30	2,911	8.408/Q1
10	International Journal of Molecular Sciences	99	1.15%	18	1,101	6.208/Q1

IF, Impact Factor; JIF Quartile, Journal Impact Factor.

At the same time, the top five publishers arranged in the order of the number of publications were sorted in **Supplementary Table 4**. Springer Nature was the most popular publisher, with 2,166 publications from 2013 to 2022, which accounted for more than a quarter of all the publications (25.26%).

In terms of research areas, 93 fields were covered. The top 10 fields rated in the number of publications were listed in **Supplementary Figure 2**. Oncology was the most represented area on account of the volume of publications (n=4,376). Besides, Genetics Heredity (n=1,182) and Biochemistry Molecular Biology (n=718) were the second and third active research areas in this knowledge domain, respectively.

### 3.5 Authors analysis

**Figure 4:**
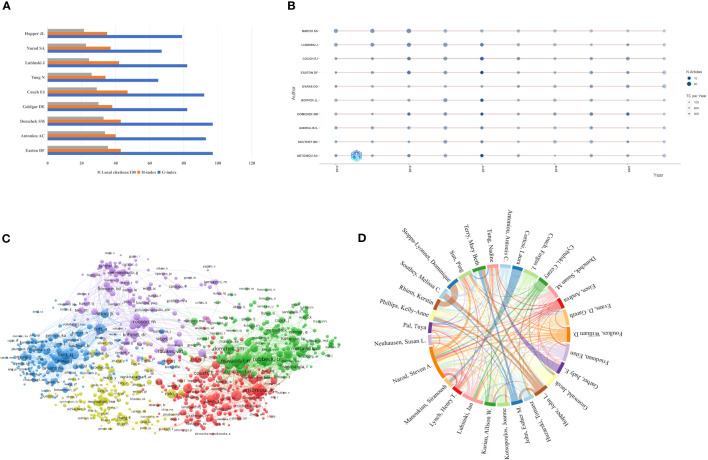
Visualization of authors’ analysis. **(A)** Visualization of the top 10 authors with the highest local impact. **(B)** Top 10 authors’ production over time. **(C)** Network map of co-citations authors. **(D)** The collaboration network of authors.

There were 42,542 authors in the author list who were working on *BRCA*-associated breast cancer research, and **Table 4** enumerates a list of the top 10 highest-yielding authors. By and large, five authors had authored over 100. The most relevant author was Narod SA from the University of Toronto, with publications 148. Narod SA, Lubinski J (n=138), and Couch FJ (n=117) ranked in the top three of the table, whereas Domchek SM (n=97) had the highest total citations (citations=9,662) of the ten authors. Among the top 10 most productive authors, their professions vary, including professors, genetic epidemiologists, oncologists, molecular biologists, and molecular geneticists. When analyzed based on their research content, these authors can be roughly categorized into three fields: I. The research focused on cancer genetics, including Lubinski J, Couch FJ, Evans DG, Easton DF, Andrulis IL, and Southey MC. II. Research related to cancer or genetic epidemiology includes Evans DG, Easton DF, and Hopper JL. III. Research on cancer risk assessment and prediction includes Domchek SM and Antoniou AC. H-index was created by Jorge E Hirsch, building on the number of publications and citations. According to Hirsch, the H-index is defined as: “A scientist has index h if h of his or her Np papers have at least H citations each and the other (Np – H) papers have ≤H citations each.” (26). G-index makes up for the defect of the H-index by taking into account the citation scores (27). Generally speaking, H-index can be used to evaluate the quantity and level of academic output of researchers, whilst G-index gives more weight to highly cited articles. Couch FJ (H-index=47), Easton DF (H-index=43), and Domchek SM (H-index=43) were the top three authors ranked by H-index. Meanwhile, Easton DF (G-index=97), Domchek SM (G-index=97), and Antoniou AC (G-index=93) had the highest G-index, which was consistent with the results ranked by total citations. Considering different career lengths, the M-index serves as a remedy to correct temporal cues, helping to identify truly successful researchers. Using H-index divided by year, we got a new index called M-index (28). Except for some authors without starting year, Couch FJ was the only author who obtained an M-index over four. In order to compare different authors visually, we also presented the number of publications, H-index, and G-index in a histogram shown in **Figure 4A**. To investigate the production over time, Bibliometrix was applied to obtain a timeline view of the top ten most yielding authors. As shown in **Figure 4B**, the node size represents the number of documents (N. Documents), and the shade of the color signifies the total number of citations (TC). It can be clearly seen that these ten authors published articles continuously throughout 2013-2022.

**Table 4:** Top 10 most productive authors in the research field of *BRCA* associated with breast cancer.

Rank	Author	Publications	Country/Region	Institution	Total Citations	H-index	G-index	M-index (Start Year)
1	Narod SA	148	Canada	University of Toronto (public)	5,128	37	67	—
2	Lubinski J	138	Poland	Pomeranian Academy of Medicine (public)	7,068	42	82	3.818 (2013)
3	Couch FJ	117	USA	University of Pennsylvania (private)	8,635	47	92	4.273 (2013)
4	Evans DG	109	United Kingdom	University of Manchester (public)	4,319	29	64	—
5	Easton DF	107	United Kingdom	University of Cambridge (public)	9,518	43	97	3.909 (2013)
6	Hopper JL	99	Australia	The University of Melbourne (public)	6,382	35	79	3.182 (2013)
7	Domchek SM	97	USA	University of Pennsylvania (private)	9,662	43	97	—
8	Andrulis IL	95	Canada	University of Toronto (public)	5,947	35	76	3.182 (2013)
9	Antoniou AC	93	United Kingdom	University of Cambridge (public)	8,696	40	93	—
9	Southey MC	93	Australia	University of Melbourne (public)	5,339	33	72	3.000 (2013)
10	Neuhausen SL	92	USA	University of Minnesota (public)	5,584	37	74	—

Co-citation analysis measures the relationship between nodes according to the number of nodes cited by the same publication (29). Author Co-Citation Analysis can visually identify and analyze scientific communities within subject areas (30). To excavate the relationship between the co-cited authors, we applied VOSviewer to generate a network map. In VOSviewer, the minimum number of citations of an author was limited to 20, and 3,285 met the threshold. There were five clusters in **Figure 4C**, which were cluster 1 (red color), cluster 2 (green color), cluster 3 (blue color), cluster 4 (yellow color), and cluster 5 (purple color). The size of the circle reflects the total link strength of different co-cited authors, and the distance between circles denotes the strength of their relationship. The representative co-cited authors of cluster 1 were Antoniou AC and Couch FJ, while in cluster 2 were Rebbeck TR and Domchek SM. Tutt A, Miki Y, and Robson M were the representative co-cited authors of the other three clusters, respectively. Furthermore, the most represented authors were Antoniou AC (total link strength = 37,524), Rebbeck TR (total link strength = 35,071), Tutt A (total link strength = 28,076), Lord CJ (total link strength =26,926), and Domchek SM (total line strength = 26,817) based on the total link strength. Among the top ten authors with the strongest total link strength, four authors were located in cluster 2.

**Figure 4D** exhibits the cooperation relationship between the most productive authors. Similarly, the size of the color block represents the number of publications, and the thickness of the line between authors reflects the number of articles they co-authored together. Narod SA (total link strength=336), Lubinski J (total link strength=296), Sun Ping (total link strength=229), as well as Gronwald J (total link strength=226) were the four authors whose total link strength exceeded 200. Besides, it was obvious that Narod SA worked most closely with Lubinski J.

### 3.6 Articles and references analysis

Researchers use citations to track the evolution of a concept through time and to choose which of a large list of papers may be the most valuable to their study (31). Besides, the local citation score (LCS) refers to the number of document citations in the local data set. It is evident that those highly cited articles can offer insightful information about scientific advances (32). All of the publications were arranged in descending order of LCS by HistCite in order to filter out the most valuable ones, as shown in **Table 5**. In June 2013, Mavaddat N et al. (LCS=357) indicated that breast cancer and contralateral breast cancer are more likely for *BRCA1* and *BRCA2* carriers through a prospective study. Concretely speaking, the estimate of *BRCA1* breast cancer incidence is 8.7‰ in the population of 20 to 29 years, while it rises to 36.1‰ when in the age group of 50 to 59 years. Moreover, the incidence rate of *BRCA2*-mutant breast cancer peaked in the population year from 40 to 49 forecast from the study (33). Antoniou AC et al. (LCS=322) studied the role of *BRCA2*-interacting protein called partner and localizer of *BRCA2* (*PALB2*) in breast cancer. They uncovered that the *PALB2* mutation female carriers have an eight to nine-fold risk of breast cancer compared with the general population in 2014 August (34). Kaufman B et al. (LCS=291) investigated the efficacy and safety of Olaparib in germline *BRCA1/2* (*gBRCA1/2*)-mutant breast cancer. After Olaparib, the breast cancer response rate was 12.9%, and the overall survival was up to 11 months. It was found that serious adverse effects occurred in 25.8% of breast cancer patients, including hyperbilirubinemia, anemia, gastroesophageal reflux, nausea, and so on (35). In February 2015, Couch FJ et al. (LCS=256) assessed the mutations in *BRCA* in triple-negative breast cancer (TNBC) through DNA sequencing. Among 1,824 patients with TNBC, mutations in the *BRCA1* (8.5%) and *BRCA2* (2.7%) genes were found in 11.2% of the population. Besides, *BRCA* carriers’ patients in TNBC have higher-grade tumors than the general population (36). In the June of the same year, Easton DF et al. (LCS=307) reviewed the sequencing technology in breast cancer-associated genes, such as *BRCA1*, *BRCA2*, *TP53*, *PALB2*, and so on (37). In February 2016, Lord CJ et al. (LCS=253) proposed a concept called BRCAness, which exists when a homologous recombination repair (HRR) defect is present in a tumor in the absence of a *gBRCA1/2* mutation. The article focused on the biomarkers and cancer histologies associated with BRCAness and their potential clinical utility. The phenotype of *BRCA1/2* mutation can serve as a prognostic tool and help perform the individual-based treatment (38). After ten months, Mirza MR et al. (LCS=240) reported the efficacy and adverse effects of Niraparib maintenance therapy in patients with platinum-sensitive, recurrent ovarian cancer. The results showed that the median length of progression-free survival was considerably longer in individuals receiving Niraparib than in those getting a placebo, regardless of the presence or absence of *gBRCA* mutations (39). In June 2017, Robson M et al. (LCS=567) found that Olaparib monotherapy was highly beneficial for patients with human epidermal growth factor receptor 2 (*HER2*)-negative metastatic breast cancer and a *gBRCA* mutation, extending progression-free survival and reducing the chance of disease progression or death (40). Kuchenbaecker KB et al. (LCS=742) estimated the age-specific breast cancer risk in the *BRCA1/2* carriers from a prospective cohort. Results displayed that the cumulative risk of breast cancer by age 80 is 72% for *BRCA1* carriers and 69% for *BRCA2* carriers. The risk of breast cancer increases with the number of first and second-degree relatives diagnosed with *BRCA1* and breast cancer (41). In 2018, Litton JK (LCS=365) et al. found that Talazoparib offered a sizable improvement in terms of progression-free survival when compared to conventional chemotherapy among *gBRCA1/2* mutation patients (42).

**Table 5:** The top 10 articles with the most local citation scores in the research field of *BRCA* associated with breast cancer.

Rank	Title	First Author, Year	Journal	LC	GC	LC/GC ratio (%)
1	Risks of Breast, Ovarian, and Contralateral Breast Cancer for BRCA1 and BRCA2 Mutation Carriers	Kuchenbaecker KB, 2017	Jama-Journal of the American Medical Association	742	1,227	60.47
2	Olaparib for Metastatic Breast Cancer in Patients with a Germline BRCA Mutation	Robson M, 2017	New England Journal of Medicine	567	1,507	37.62
3	Talazoparib in Patients with Advanced Breast Cancer and a Germline BRCA Mutation	Litton JK, 2018	New England Journal of Medicine	365	926	39.42
4	Cancer Risks for BRCA1 and BRCA2 Mutation Carriers: Results from Prospective Analysis of EMBRACE	Mavaddat N, 2013	Journal of the National Cancer Institute	357	570	62.63
5	Breast-Cancer Risk in Families with Mutations in PALB2	Antoniou AC, 2014	New England Journal of Medicine	322	546	58.97
6	Gene-Panel Sequencing and the Prediction of Breast-Cancer Risk	Easton DF, 2015	New England Journal of Medicine	307	541	56.75
7	Olaparib Monotherapy in Patients with Advanced Cancer and a Germline BRCA1/2 Mutation	Kaufman B, 2015	Journal of Clinical Oncology	291	1,201	24.23
8	Inherited Mutations in 17 Breast Cancer Susceptibility Genes Among a Large Triple-Negative Breast Cancer Cohort Unselected for Family History of Breast Cancer	Couch FJ, 2015	Journal of Clinical Oncology	256	414	61.84
9	BRCAness revisited	Lord CJ, 2016	Nature Reviews Cancer	253	729	34.71
10	Niraparib Maintenance Therapy in Platinum-Sensitive, Recurrent Ovarian Cancer	Mirza MR, 2016	New England Journal of Medicine	240	1,374	17.47

LC, local citations; GC, global citations.

Compared with LCS, the index of local cited references (LCR) can quickly figure out the most relevant articles in the research direction. In **Table 6**, we elicited the top ten most locally cited references. Mekonnen N et al. reviewed the mechanism and tactics for overcoming PARPi resistance. By lowering *BRCA1*’s ability to repair DNA, the high expression of RING domain-deficient *BRCA1* proteins in breast cancer cell lines increases resistance to cisplatin and PARPi, while deletion mutations in *BRCA2* would result in resistance to PARPi (11). Russi M et al. introduced the functional characteristics and structural insights of *BRCA1* and the *BRCA1* mutations on cancer progression (43). Slade D et al. provided an overview of the targeting DNA damage response drugs PARPi and poly-adenosine diphosphate ribose glycohydrolase (PARG) and illustrated the clinical performance of four kinds of PARPi drugs (Olaparib, Rucaparib, Niraparib, and Talazoparib) (44). By conducting a review of the clinical data from large cohorts, Gianni P et al. summarized the role of Fanconi anemia-associated genes in sporadic and familial breast cancer (45). Yordanova M et al. also indicated that the application of PARPi in monotherapy or combination therapy has great potential in TNBC patients (46). Trusler O et al. presented *BRCA1* and *BRCA2* cancer biology and then introduced the relevant disease modelling systems, thus, providing guidance in drug design (47). Bellcross CA et al. provided an update on hereditary breast cancer associated with pathogenic variants in *BRCA1/2* (48). Menezes MCS et al. reviewed the application of PARPi drugs like Olaparib and Talazoparib and a series of biomarkers of response to PARPi drugs. At the same time, the authors pointed out that the future work direction is to identify the biomarker of response to PARPi (49). Cortesi L et al. summarized an update on oral PARPi in breast cancer treatment and gave a reference for identifying the population who would suffer benefits from PARPi (50). Dias MP et al. summed up the underlying resistance to PARPi and possible strategies such as combinations with chemotherapies, targeting the acquired vulnerabilities associated with resistance to PARPi, or suppressing genomic instability (51).

**Table 6:** Top 10 most locally cited references in the research field of *BRCA* associated with breast cancer.

Rank	Title	Journal	First Authors, Year	LCR
1	Homologous Recombination Deficiency in Ovarian, Breast, Colorectal, Pancreatic, Non-Small Cell Lung and ProStates Cancers, and the Mechanisms of Resistance to PARP Inhibitors	Frontiers in Oncology	Mekonnen N, 2022	72
2	The fellowship of the RING: BRCA1, its partner BARD1 and their liaison in DNA repair and cancer	Pharmacology & Therapeutics	Russi M, 2022	58
3	PARP and PARG inhibitors in cancer treatment	Genes & Development	Slade D, 2020	57
4	The Fanconi anemia pathway and Breast Cancer: A comprehensive review of clinical data	Clinical Breast Cancer	Gianni P, 2022	52
5	Expanding the Use of PARP Inhibitors as Monotherapy and in Combination in Triple-Negative Breast Cancer	Pharmaceuticals	Yordanova M, 2021	49
6	BRCA1 and BRCA2 associated breast cancer and the roles of current modelling systems in drug discovery	Biochimica et Biophysica Acta-Reviews on Cancer	Trusler O, 2021	48
7	Hereditary Breast and Ovarian Cancer An Updated Primer for OB/GYNs	Obstetrics and Gynecology Clinics of North America	Bellcross CA, 2022	47
8	PARP Inhibitors for Breast Cancer: Germline BRCA1/2 and Beyond	Cancers	Menezes MCS, 2022	47
9	An Overview of PARP Inhibitors for the Treatment of Breast Cancer	Targeted Oncology	Cortesi L, 2021	44
10	Understanding and overcoming resistance to PARP inhibitors in cancer therapy	Nature Reviews Clinical Oncology	Dias MP, 2021	44

LCR, local cited references.

**Figure 5:**
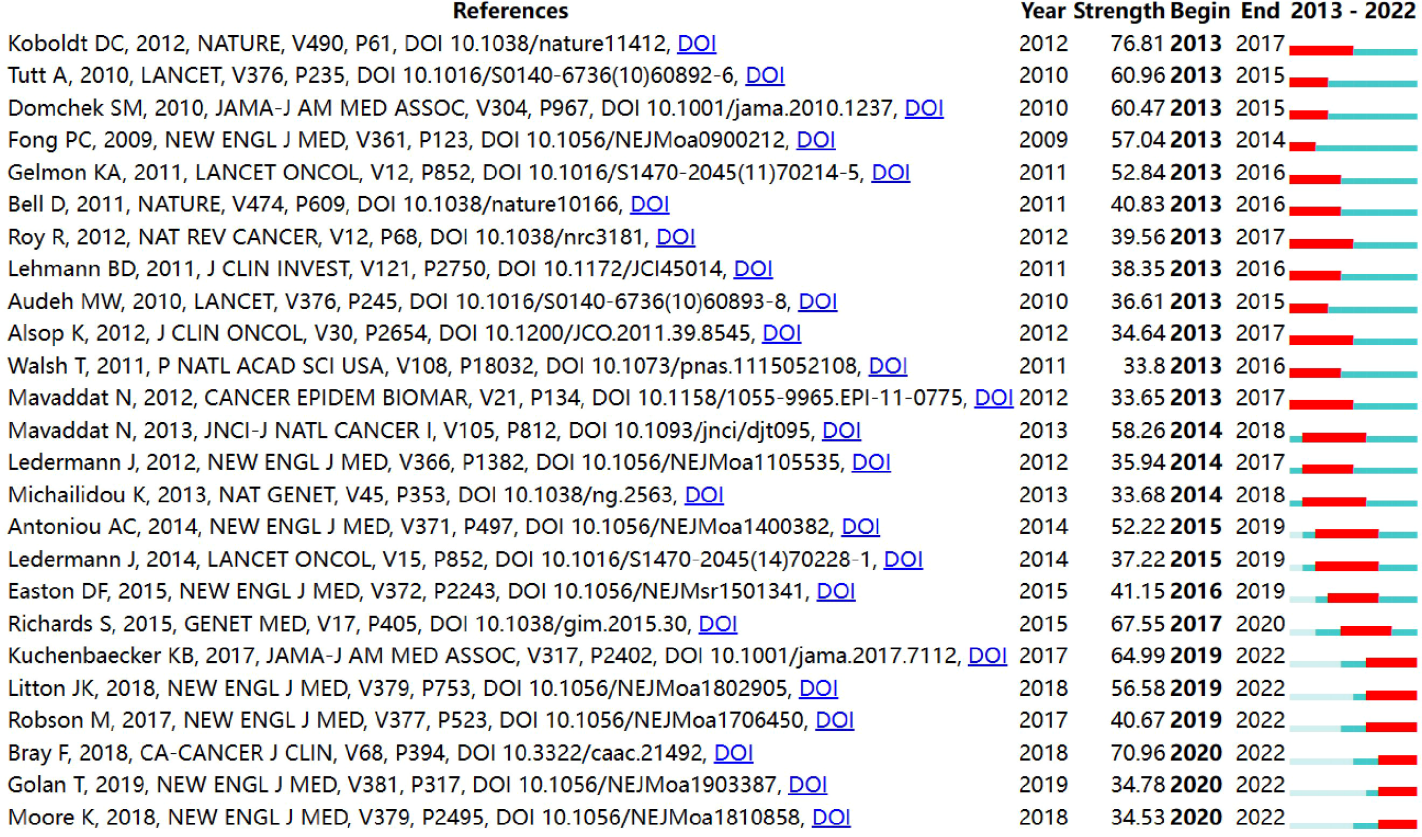
Top 25 references with the strongest citation bursts.

By CiteSpace, we got a list of the top 25 references with the strongest citation bursts, as shown in **Figure 5**. In the figure, the red bar denotes the actively cited period, and the 25 references can be divided into three periods by burst year. In recent years, the articles published by Kuchenbaecker KB et al. (strength=64.99) and Litton JK et al. (strength=56.58) were previously mentioned (41, 42). Bray F et al. (strength=70.96) presented an estimate of various kinds of cancer incidence worldwide (52), thus, providing research background for the *BRCA* in breast cancer.

### 3.7 Keywords analysis

**Figure 6:**
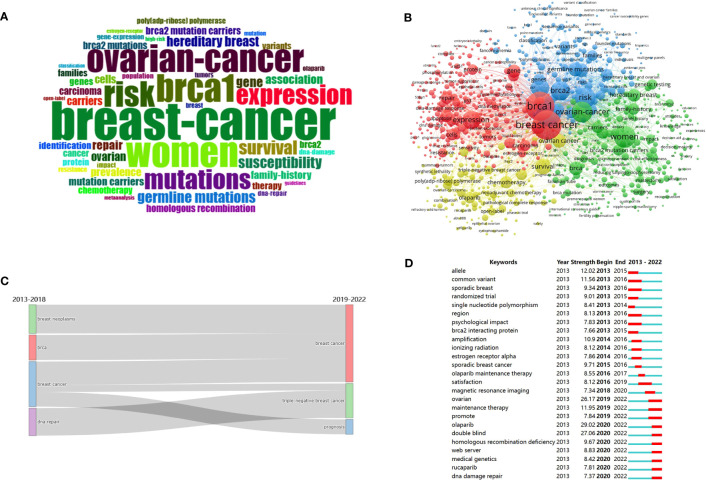
Visualization of keywords analysis. **(A)** Keyword cloud of the retrieved articles. **(B)** Co-occurrence analysis of keywords. **(C)** Sankey diagram of the evolution of the keywords. **(D)** Top 25 keywords with the strongest citations bursts.

Keyword analysis is a process of analyzing articles that allows us to identify the hotspots and trends in certain fields explicitly (53). Bibliometrix was applied to visualize the occurrence and frequency of keywords. A total of 9,568 keywords proposed by the authors in the article were included in this study. **Figure 6A** is a keyword cloud of the retrieved articles, and the font size is in direct proportion to its frequency. From the figure, we can glance at essential words over the past ten years. Firstly, keywords ranked in the top three were “breast cancer”, “women”, and “*brca1*”. Next were closely followed by “ovarian cancer”, “risk”, “mutations”, “expression”, and “survival”. Moreover, the corresponding proportion of the top 25 frequent keywords was shown in **Supplementary Figure 3**, and the frequency of occurrence is described by the size of the color block.

Two keywords emerge together in a research paper called co-occurrence keywords, they generally have some correlation, and this correlation can be expressed by the frequency of co-occurrence. The distance between the nodes depends on their co-occurrence frequency. A co-occurrence network between keywords is displayed in **Figure 6B**, and the sum of occurrence times is presented in the node size. The keywords are divided into five clusters and represented by a color. They are cluster 1 (red color), cluster 2 (green color), cluster 3 (blue color), cluster 4 (yellow color), and cluster 5 (purple color). Those keywords with total link strength of more than 2,500 were selected as representative words, as shown in **Table 7**.

**Table 7:** Cluster of keywords in co-occurrence analysis.

Cluster	Color	Representative Keywords
1	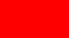	brca1, breast cancer, expression, gene, homologous recombination, repair, cancer, carcinoma, identification, cells, prognosis
2	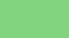	women, ovarian cancer, brca, hereditary breast, family-history, carriers, mutation carriers, brca2 mutation carriers, genetic testing
3	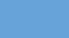	risk, brca2, mutations, germline mutations, susceptibility, prevalence, association, ovarian, brca2 mutations, genes, mutation, families, variants, poly(adp-ribose) polymerase
4	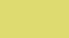	survival, chemotherapy, Olaparib, therapy
5	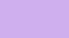	features

**Figure 6C** illustrates the time flow of keywords in *BRCA* associated with breast cancer during 2013-2022. The Sankey diagram divides the decade into two periods: the color block on the left represents the keywords during 2013-2018, while the left block denotes keywords from 2019-2022. It mainly shows the shift from “DNA repair” and “breast cancer” to “triple-negative breast cancer”, and “breast cancer” shifted into “prognosis”.

Citation burst words refer to those cited frequently over a period. In **Figure 6D**, the top 25 keywords with the strongest citation bursts were extracted using CiteSpace. As seen in the figure, the 25 keywords have gone through three periods according to the citation burst period. The first period: some keywords actively involved in former years, such as “allele”, “common variant”, “sporadic breast”, “randomized trial”, “single nucleotide polymorphism”, “region”, “psychological impact”, “*brca2* interacting protein”. The intermediate stage contained “amplification”, “ionizing radiation”, “estrogen receptor alpha”, “sporadic breast cancer”, “olaparib maintenance therapy”, “satisfaction”, and “magnetic resonance imaging”. Others actively involved in recent years served as the third period, including “ovarian”, “maintenance therapy”, “promote”, “olaparib”, “double-blind”, “homologous recombination deficiency”, “web server”, “medical genetics”, “rucaparib”, and “dna damage repair”. Of the 25 keywords, “olaparib” (strength=29.02), “double-blind” (strength=27.06), and “ovarian” (strength=26.17) were the three keywords with the strongest strength.

**Figure 7:**
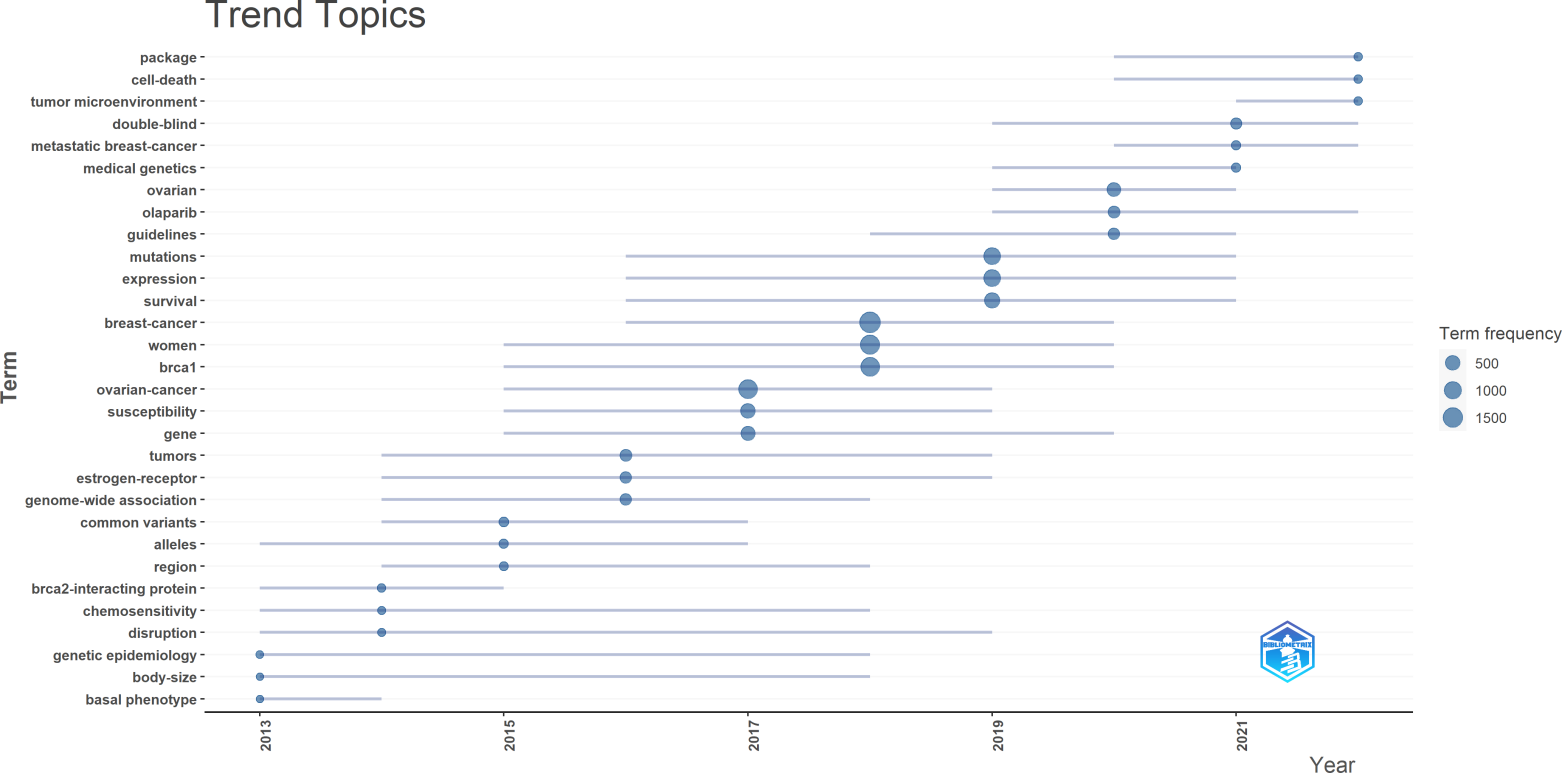
Trend topics from 2013-2022 in the research field of *BRCA* associated with breast cancer.

The trend topics in the study of *BRCA* in breast cancer were investigated in order to excavate the research details. In **Figure 7**, the size of the node represents the occurrence frequency of a term, while the grey line indicates the duration time. “Breast cancer”, “women”, and “*brca 1*” were the most relevant terms, and “ovarian cancer” followed closely. It was worth noting that “olaparib”, “metastatic breast cancer”, “double-blind”, “tumor microenvironment”, “cell death”, and “package” emerged in recent years and behaved actively continuously until 2022.

## 4 Discussion

We carried out a scientometric analysis on 8,576 publications correlated with *BRCA* as well as breast cancer during the past decade through bibliometric, VOSviewer, CiteSpace, and HistCite Pro. In the last decade, the general trend of the number of scientific outputs had a steady rise. Production of articles had grown at an average annual rate of 6.57% and peaked in 2021, only with a slight negative growth rate in 2017 and 2022. Beyond that, the year with the relatively high growth rate was 2021 and 2015, which signified a sharp increase in attention in the research field. The occurrence of ground-breaking publications with high LCS, as shown in **Table 5**, was the main reason for the surge in the growth rate. It can be concluded from the rising trend that this research field is in full swing. Furthermore, the high average citations per article mean that publishing research on *BRCA* in breast cancer in high-quality journals is not a challenge. It can be preliminarily inferred from **Figure 2B** that the publications in 2017 could have a significant influence on the study of *BRCA*-mutant breast cancer. During the past decade, the mean total citations per article decreased year by year to a first approximation. Although the number of citations is an important indicator to measure the academic impact of a publication, it should be noted that due to the time factor, earlier studies have more possibility to be cited more frequently than more recently published studies, which means the academic impact of earlier studies may not be stronger than later studies (54, 55).

In countries/regions analysis, the USA significantly exceeded nearly a thousand more than the second-ranked country. Additionally, its average article citations were 32.19, and it ranked second in the top ten countries. From the data above, we can firmly conclude that the USA was ahead of others in the research field. According to the American Cancer Society’s estimates, about 13% of women from the USA would develop breast cancer, which may be the underlying reason for the great number of publications in the USA. In particular, the only developing country appearing on the list was China, indicating that China went forefront among developing countries in the study of *BRCA*-mutant breast cancer. Though China followed the USA, it held a relatively low average article citations of 11.85. Behind the phenomenon, a probable explanation is that China academics focus more on quantity but not quality (56). In general, the research on *BRCA* had been heavily skewed towards developed countries like the USA, so the occurrence of China was encouraging to developing countries. Besides, China was the country that worked the tightest with the USA. In the collaboration network, the total link strength of the USA was 3,047 and ranked first in the world. Given the evidence above, the USA was the central country for publications. From the institutions’ analysis, the University of Toronto ranked first among the top 10 institutions in the number of articles. In the list, there were four institutions in the USA, which was consistent with the countries’ analysis. It was a pity that no institutions were located in developing countries. Besides, the University of Toronto held the strongest link strength and frequently cooperated with Pomeranian Medical University.

From the journals’ analysis, *Breast Cancer Research and Treatment* was the most popular journal, while *Breast Cancer Research* ranked first in IF in the research of *BRCA* associated with breast cancer. According to Bradford’s Law, a list of 25 core journals was presented in **Supplementary Table 3**, which was the main force in contributing to the important publications. Journal analysis may help offer the researchers guidance in choosing journals. In addition, the most relevant publisher was Springer Nature which accounted for nearly one-quarter (25.26%). Oncology, Genetics Heredity, and Biochemistry Molecular Biology were key components of the research areas. It can be drawn out that the gene and molecular mechanism of *BRCA* in breast cancer is the central point at the moment.

In the authors’ analysis, we applied the number and citations of publications to assess an author. Narod SA from the University of Toronto led the way in the sum of publications. Couch FJ had the highest H-index and M-index from the University of Pennsylvania, while Easton DF and Domchek SM had the highest G-index. Therefore, more attention should be attached to Couch FJ, Easton DF, and Domchek SM to grasp the important advances in the field. Besides, we can know that these ten prolific authors have been sticking in this field for a long time and continue to produce articles from **Figure 4B**. It also reflects that the study about *BRCA* in breast cancer still has much to explore from another perspective.

Amongst the 8,576 retrieved articles, we used HistCite to identify the articles with the highest LCS and the LCR. In **Table 5**, the research direction of the articles can be divided into three dimensions: I. Conduct a prospective cohort on the patients with *BRCA*-mutant breast cancer to predict the incidence in different age groups (33, 41). II. Review the advances in breast cancer-associated genes, including the sequencing technology, evaluation of *BRCA* mutations in specific breast cancer, and relevant biomarkers (36–38). III. Explore the efficacy and safety of PARPi drugs like Olaparib and Talazoparib in *BRCA1/2*-mutant breast cancer (35, 42). According to the LCS analysis, researchers can grasp the key development in the field of *BRCA* associated with breast cancer. Furthermore, half of the publications in the list were published in the journal *New England Journal of Medicine*. Therefore, more attention should be attached to this journal on relevant topics. In the most locally cited references shown in **Table 6**, some articles chiefly discussed the mutations, function, and carcinoma biology of *BRCA1/2* (43, 47, 48). On the other hand, there were six articles concerning PARPi drugs, including the clinical performance, the mechanism of resistance and coping strategies, relevant biomarkers, identification of the target population, and so on (11, 44, 46, 49–51). References with strong citation bursts refer to articles with a surge in citations after publication, which indicates that researchers pay great attention to related topics. From **Figure 5**, the age-specific risk of breast cancer in *BRCA* carriers and the therapeutic effect of Talazoparib are the two new hotspots in recent years (41, 42).

Keywords are the core of a paper summary. The paper keyword analysis can be a glimpse of the topic of the article. In the keywords cloud, “risk”, “mutations”, “expression”, and “survival” frequently occurred in the 8,576 articles except for the entry terms, which denoted that risk estimates and survival effects of *BRCA* expression and mutations were the main research direction over the past decade. In **Figure 6B**, cluster 1, shown in red, contained “*brca1*”, “breast cancer”, “expression”, “gene”, “homologous recombination”, “repair”, and “identification”, indicating this cluster focuses on the underlying mechanism of *BRCA* mutation-associated homologous recombination (HR) and relevant, targeted drugs. Cluster 2 in green includes “hereditary breast cancer”, “family history”, “carriers”, and “genetic testing”, and the main emphasis is on genetic factors in breast cancer. Cluster 3 in blue comprises “risk”, “*brca2*”, “mutations”, “germline mutations”, “susceptibility”, “prevalence”, “association”, and “variants”, paying great attention to the *BRCA* mutations in breast cancer and estimates on prevalence. Cluster 4, colored in yellow, consists of “survival”, “chemotherapy”, “olaparib”, and “therapy”, which mainly centred the treatment and prognosis. From the citation burst words, the shift from the early stage to recent years is clearly seen in **Figure 6D**. In the early stage, “allele” and “common variant” burst from 2013 to 2016 with relatively high strength. This stage mainly explored the mutations of breast cancer-associated genes, including *BRCA1/2*, *PALB2*, and so on, through sequencing technology and the risk of breast cancer (57–63). In the intermediate stage, “amplification” and “sporadic breast cancer” burst from 2014 to 2016. Amplification technology such as multiplex ligation-dependent probe amplification has been frequently used to detect the *BRCA* mutations and other relevant genes in breast cancer patients and help identify the therapeutic effect of breast cancer surgical specimens (64–67). While the latter one reflected in the exploration of the *BRCA1* role and mutations in sporadic breast cancer (68–73). In recent years, “olaparib” and “double-blind” burst from 2020 to 2022, denoting the evaluation of the efficacy and safety of Olaparib in both monotherapy and combination therapy among patients with *BRCA1/2*-mutant breast cancer (74–77). In brief, the shift from the early years to the second period reflected that the study of pathogenesis went from shallow to deep, like more specific cancer types; And people have put emphasis on the clinical trials of targeted drugs in recent years. There would be a definite need to develop drugs and conduct clinical trials based on the former research results. In the case of normal cells, DNA repair is responsible for the integrity and stability of DNA, among which the HRR is regarded as the most accurate and high-fidelity DNA damage repair system. While *BRCA1* and *BRCA2* are the most common genes involved in HRR pathways, homologous recombination repair (HRD) may arise if *BRCA1/2* mutate. Moreover, PARPi is primarily targeted at *PARP1*, a DNA damage sensor and signal transducer. It could detect the SSB and double-strand break (DSB) (78) and play an important role in stabilizing the replication fork (79, 80). Breast cancer cells combined with *BRCA1/2*-mutant and PARPi treatment would lead to the accumulation of DSB and bring in synthetic lethality (81–83). In addition to influencing the DNA repair pathways, some studies have found that PARPi could generate anti-tumor effects through immunoregulation. PARPi Talazoparib could promote the amount of peritoneal CD8+ T cells and natural killer cells, thus participating in anti-tumor (84). Another similar case is that an intact immune system, especially CD 8+, is required for the best Olaparib response in animal tumor models (85). Currently, PARPi Olaparib and Talazoparib have gained approvals for the therapy of metastatic or locally advanced *gBRCA*-mutant or *HER-2* negative breast cancer from the Food and Drug Administration after the basis of Phase III clinical trials (40, 42). It is noteworthy that a history of prior exposure to platinum chemotherapy may decrease the response rate among patients with advanced and *gBRCA1/2-*mutant breast cancer (35). Except for the PARPi mentioned above, a trial has proved that Veliparib combined with platinum therapy would prolong the non-prognosis survival in *HER-2* negative advanced and *gBRCA1/2-*mutant breast cancer (86). However, compared with the paclitaxel plus carboplatin group, there was no significant difference in the pathologic complete response rate of the combined treatment effect of paclitaxel, carboplatin and Veliparib (87). Since the promising future of PARPi, identifying PARPi-sensitive breast cancer patients and expanding the targeted population would be an important research goal. At first, *BRCA1* and *BRCA2* mutations were identified as biomarkers in HRD, but there still exists some HRD cancer without *BRCA1/2* mutations (88), indicating that some other HR-related genes would potentially affect PARPi sensitivity. Clinical trial TBCRC 048 put forward that germline *PALB2* or somatic *BRCA1/2*-mutant breast cancer responds well to PARPi (89). Besides accumulation of *γH2AX* and *RAD51 recombinase* (*RAD51*) nuclear foci formation, the level of loss of heterozygosity events or telomeric allele imbalance could serve as predictive biomarkers for the identification of who benefit from PARPi therapy (90). Moreover, other DNA damage response genes like checkpoint kinase 1, ataxia telangiectasia and Rad3-related, cyclin-dependent kinase 12 and so on show synthetic lethality under PARPi therapy (44, 83, 91). In brief, searching for more sensitive and effective HRD biomarkers is a bright research direction. With the expansion of the PARPi therapeutic scope, further research is needed to identify emerging resistance mechanisms. The widely studied mechanism of PARPi resistance is the restoration of the HR function, and it appears when secondary mutations on HR-related genes like *BRCA1/2* and *RAD51C/D*. Secondly, *BRCA1*, *BRCA 2*, and *PARP1* may increase the stabilization of the replication fork. Another alternative mechanism is the mutations in the DNA-binding domains of *PARP1*: such as the loss of *PARG* could decrease the *PARP1* trapping and lead to PARPi resistance. Therefore, PARGi can be used in addition to PARPi to reduce resistance (90, 92). Furthermore, L. Tobalina et al. found that most amino acid sequences encoded by *BRCA1* and *BRCA2* exon 11 are essential components of resistance to PARPi through a meta-analysis of 86 *BRCA1/2*-mutant cases. Inhibiting DNA end-joining repair pathways could prevent *BRCA1/2* from reversion (93). Other possible strategies, like the combination with cyclin-dependent kinase 1 inhibitors, androgen receptor inhibitors, dinaciclib, and immune checkpoint inhibitors, would be of some help in facilitating the PARPi treatment (11).

Translational research on BRCA-related genes in a clinical setting focuses on areas such as genetic testing, risk prediction, targeted therapies, treatment response, and risk-reducing excision. It can help guide personalized screening and treatment strategies after the identification of the benefit population (94–97). The search for additional synthetic lethality methods is also an active area (98).

Finally, it cannot be denied that there exist some deficiencies in the article. In countries analysis, however, some potential factors may carry significant weight in the production of a country. According to the website “Our World in Data,” the population of the USA is 5.69 times larger than that of Italy, which partly explains why a mere comparison of the number of publications is not comparable. Similarly, comparing developing countries with strongly developed countries can introduce more bias due to differences in levels of development. In addition, diverse publishing ethics, industrial standards, and censorship practices always result in differences in output quantity, quality, and modes of distribution around the world. In more lenient countries, editors may willingly publish bold and controversial material that generates more output and yields greater profits, albeit drawing criticism and causing controversy. Since population and publishing ethics vary from country to country, the production discrepancy deserves further exploration. The data of the study only retrieved related literature from the WoSCC exclusively and ignored some influential data sources, such as PubMed, EMBASE, SCOPUS, and so on. One reason is that some literature databases lack citations like PubMed, and another is that data from different databases cannot be merged. Moreover, language bias existed in the data collection because we merely chose articles published in English. Therefore, our data could not cover all related publications in this research field and even neglected some essential publications. Secondly, the literature analysis software VOSviewer, and CiteSpace may result in slight distinctions due to version issues. Thirdly, although citation is a widely used index, whether it can reflect academic influence remains controversial (55). We applied citations as the main assessment method may prompt some newly published influential articles being unnoticed (99). In addition, certain articles may exhibit contradictions in comparison to recently published literature, attributable to their publication date. Moreover, Open Access (OA) journals offer freely available articles, and they often rely on Article Publishing Charges (APCs) to sustain their operations and cover expenses. There are reports indicating that OA can have a significant impact on the journal impact factor of medical journals. Additionally, it has been suggested that OA can enhance the academic influence of a journal (100, 101). Furthermore, the vast majority of the open-access articles come from high-income countries (102). Regrettably, we can hardly eliminate the biases caused by APCs in the research outcomes. Ultimately, we stress that the results only demonstrate the current research trends in academia and may not accurately correspond to actual applications or impact.

## 5 Conclusion

Research on *BRCA* has spanned decades, and its function in the incidence and development of breast cancer has a bright research future. The scientometric study analyzed the relevant articles ranging from 2013 to 2022 on *BRCA* associated with breast cancer. It systematically explored the following analyses: annual growth trend of publications, countries, institutions, journals, authors, landmark articles, collaboration networks, keywords analysis, et al. In the past decade, the research on *BRCA* has transited from the molecular mechanism level to the clinical application level. Combining the articles and keywords analysis, the age-specific risk of breast cancer and the therapeutic effects of PARPi among *BRCA* carriers are the recent hotspots. Therefore, the future research direction would focus on conducting clinical trials of PARPi, including efficacy, safety, and combination therapy in the different subtypes of *BRCA1/2*-mutant breast cancer. Besides, the exploration of more sensitive and efficient biomarkers for guiding targeted therapy is in great need. The in-depth analysis of breast cancer associated with *BRCA* will provide new ideas for the *BRCA* targeting treatment and insights for relevant researchers to identify new directions. Owing to the grim situation of the high occurrence of breast cancer, a reasonable assumption is that *BRCA* would receive increasing attention in recent years.

## Data availability statement

The original contributions presented in the study are included in the article/**Supplementary Material**. Further inquiries can be directed to the corresponding authors.

## Author contributions

YH and DZ originated and formulated the research project, conducted the visual analysis of the data, completed the initial draft, and revised the manuscript in accordance with the feedback and recommendations of QY, JW, and HT. ZJ and LC retrieved the dataset from the WoS and rendered guidance on software utilization. JC, ZL, and YC undertook the task of analyzing and comprehending the research outcomes. All authors contributed to the article and approved the submitted version.
