# Supplementary material for: Global trends in BRCA-related breast cancer research from 2013 to 2022: A scientometric analysis
Source: Front Oncol. 2023 Jul 5;13:1197168. doi: 10.3389/fonc.2023.1197168 (PMC10354558; doi:10.3389/fonc.2023.1197168)
Supplement: Supplementary file 1 [file DataSheet_1.docx]

Supplementary Material

**Supplementary Table 1** | Summary of data source and descriptive statistics.

| Category | Specific Standard Requirements |
| --- | --- |
| Research database | Web of Science core collection |
| Citation indexes | SCI |
| Searching period | January 2013 to December 2022 |
| Document types | “Article” or “Review Article” |
| Language | “English” |
| Data extraction | Export with full record and cited references in plain text format |
| Sample size | 8,576 |
| H-index | 161 |
| Average annual growth rate % | 6.57 |
| Articles average age | 4.98 |
| Times Cited（self-citations） | 196,280（42,207） |
| Average citations per article | 22.89 |
| References | 179,404 |
| Author’s keywords | 10,610 |
| Authors | 42,542 |
| Authors of single-authored articles | 133 |
| Co-authors per article | 10.6 |
| International co-authorship % | 28.42% |
| Journals | 1,244 |
| Institutions | 8,555 |
| Publishers | 263 |
| Countries | 105 |

**Supplementary Table 2** | Mean annual total citations between 2013-2022 in the research field of *BRCA* associated with breast cancer.

| Year | Publications | MeanTCperArt | MeanTCperYear | Citable Years |
| --- | --- | --- | --- | --- |
| 2013 | 623 | 37.35 | 3.40 | 11 |
| 2014 | 663 | 36.43 | 3.64 | 10 |
| 2015 | 741 | 38.35 | 4.26 | 9 |
| 2016 | 796 | 35.56 | 4.44 | 8 |
| 2017 | 779 | 34.1 | 4.87 | 7 |
| 2018 | 845 | 28.21 | 4.70 | 6 |
| 2019 | 920 | 22.91 | 4.58 | 5 |
| 2020 | 989 | 13.08 | 3.27 | 4 |
| 2021 | 1,129 | 7.11 | 2.37 | 3 |
| 2022 | 1,091 | 1.25 | 0.62 | 2 |

Notes: Mean TcperArt = Mean total citations per article; MeanTCperYear = Mean total citations per year.

**Supplementary Table 3** | The core journals in the research field of *BRCA* associated with breast cancer according to Bradford’s Law.

| Rank | Journals | Publications | H-index | Total Citations | IF (2022) | Production Year Start |
| --- | --- | --- | --- | --- | --- | --- |
| 1 | Breast Cancer Research and Treatment | 350 | 39 | 6,153 | 4.624 | — |
| 2 | Cancers | 254 | 19 | 2,148 | 6.575 | 2014 |
| 3 | PLoS One | 177 | 33 | 4,048 | 3.752 | 2013 |
| 4 | BMC Cancer | 160 | 27 | 2,597 | 4.638 | 2013 |
| 5 | Scientific Reports | 154 | 24 | 2,002 | 4.996 | 2013 |
| 6 | Oncotarget | 143 | 33 | 3,537 | 4.345 | 2013 |
| 7 | Frontiers in Oncology | 140 | 17 | 1,031 | 5.738 | 2015 |
| 8 | Familial Cancer | 124 | 17 | 1,198 | 2.446 | — |
| 9 | Journal of Genetic Counseling | 104 | 21 | 1,314 | 2.717 | — |
| 10 | Breast cancer Research | 99 | 30 | 2,911 | 8.408 | 2013 |
| 11 | International Journal of Molecular Sciences | 99 | 18 | 1,101 | 6.208 | 2013 |
| 12 | International Journal of Cancer | 94 | 28 | 2,374 | 7.316 | — |
| 13 | Clinical Cancer Research | 88 | 36 | 4,416 | 13.801 | 2013 |
| 14 | Frontiers in Genetics | 82 | 12 | 431 | 4.772 | 2014 |
| 15 | Hereditary Cancer in Clinical Practice | 82 | 14 | 704 | 2.164 | 2013 |
| 16 | Breast | 81 | 19 | 1,199 | 4.254 | 2013 |
| 17 | Annals of Surgical Oncology | 76 | 22 | 1,577 | 4.339 | — |
| 18 | British Journal of Cancer | 76 | 25 | 2,459 | 9.075 | 2013 |
| 19 | Journal of Clinical Oncology | 76 | 42 | 7,625 | 50.717 | 2013 |
| 20 | Gynecologic oncology | 74 | 25 | 2,099 | 5.304 | 2013 |
| 21 | Genetics in Medicine | 68 | 29 | 2,377 | 8.864 | 2013 |
| 22 | Nature Communications | 68 | 31 | 3,239 | 17.694 | 2013 |
| 23 | Oocology Letters | 68 | 14 | 555 | 3.111 | 2013 |
| 24 | Annals of Oncology | 67 | 42 | 5,605 | 51.769 | 2013 |
| 25 | Genes | 64 | 10 | 403 | 4.141 | 2016 |

**Supplementary Table 4** | Top 5 most productive publishers in the research field of *BRCA* associated with breast cancer.

| Rank | Publishers | Publications | % of 8,576 publications |
| --- | --- | --- | --- |
| 1 | Springer Nature | 2,166 | 25.26% |
| 2 | Elsevier | 1,165 | 13.58% |
| 3 | Wiley | 863 | 10.06% |
| 4 | Mdpi | 559 | 6.52% |
| 5 | Frontiers Media Sa | 321 | 3.74% |


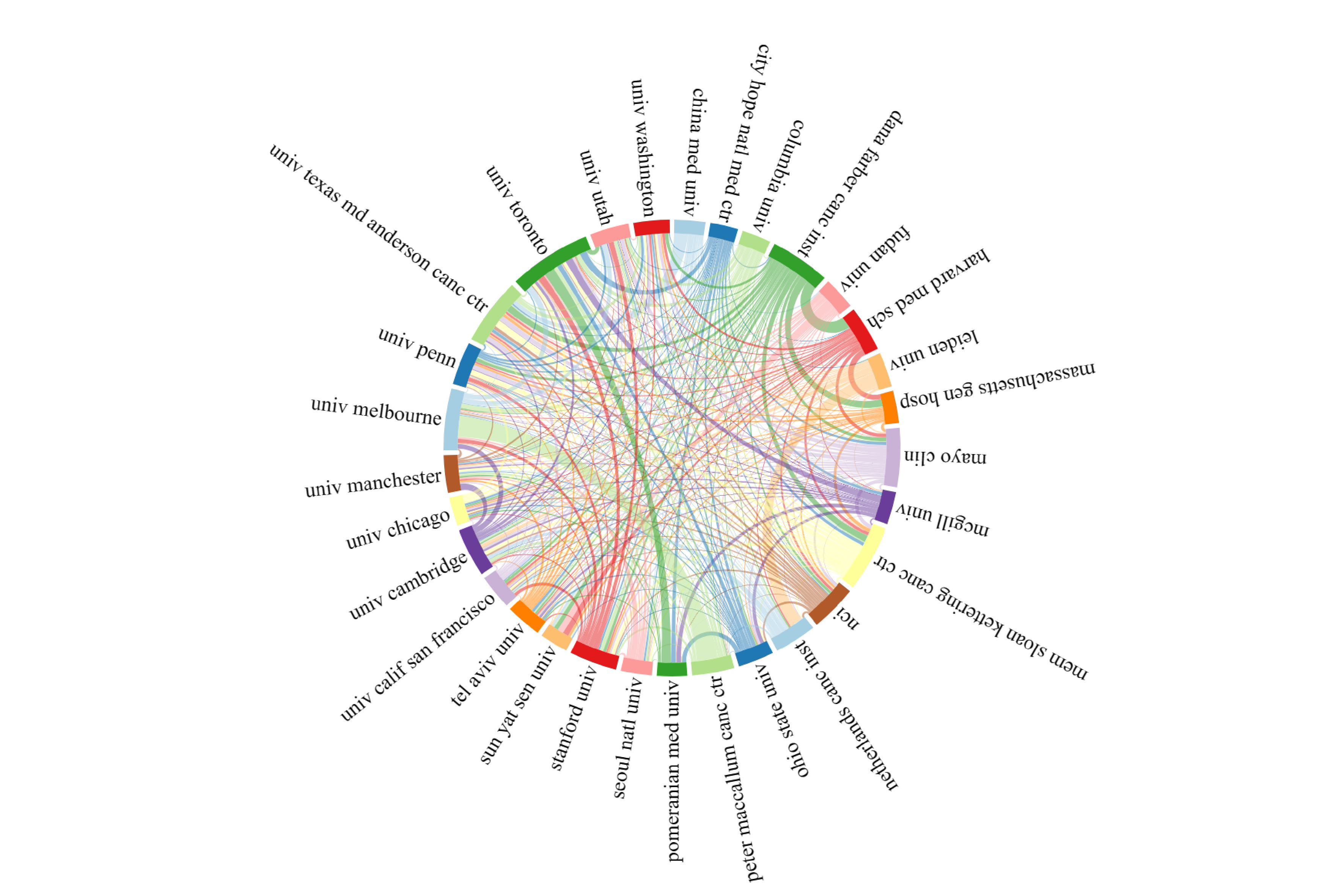


**Supplementary Figure 1** | The collaboration network between institutions.


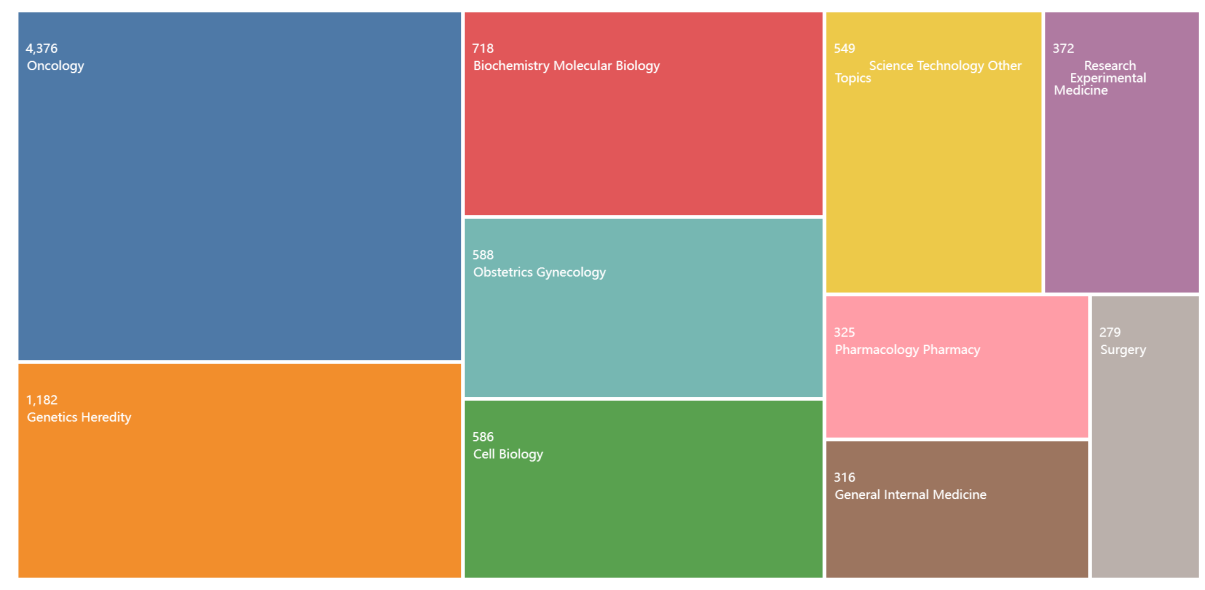


**Supplementary Figure 2** | Treemap of the top 10 most productive research areas.


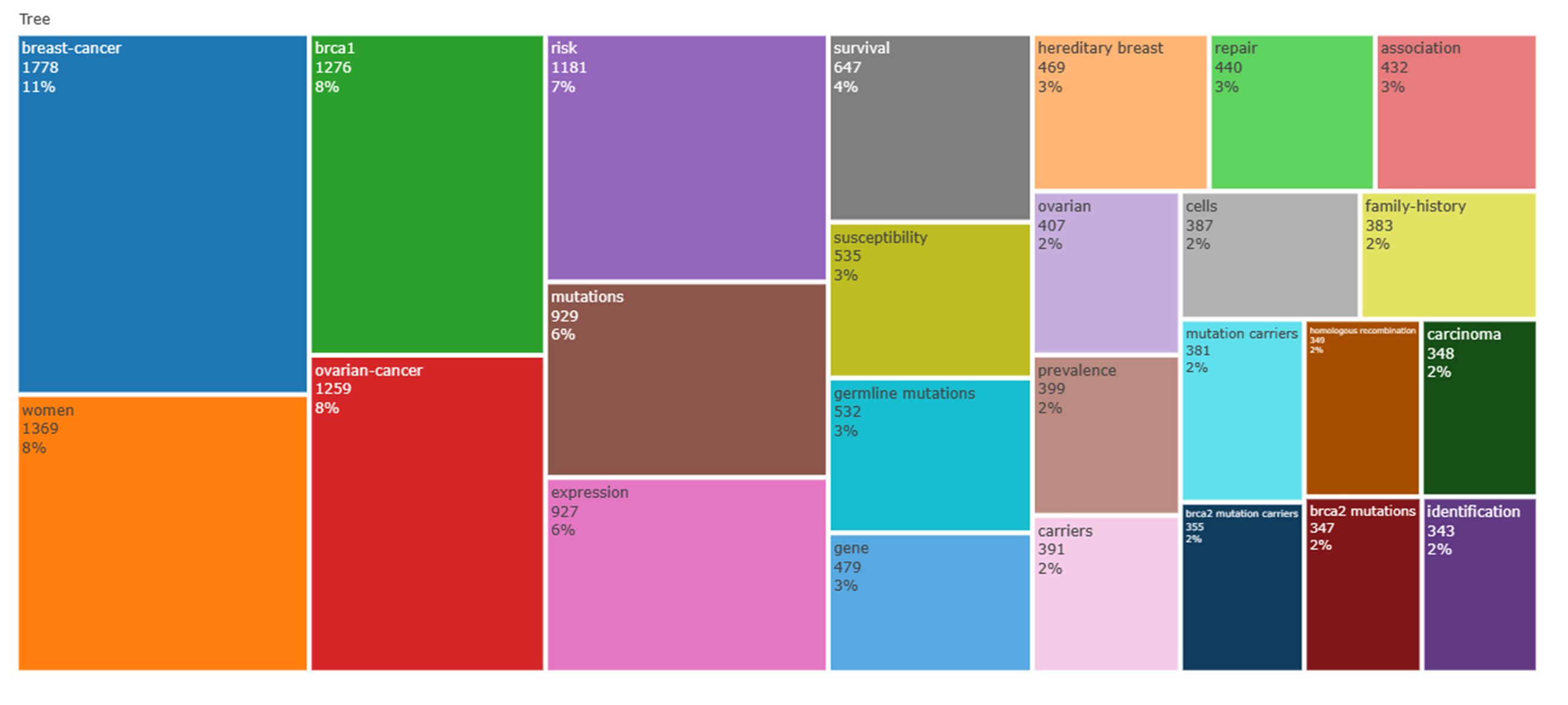


**Supplementary Figure 3** | Treemap of the corresponding proportion of keywords.
